# Strategies for the Selection and Application of Biological Scaffolds in Organ‐on‐a‐Chip Systems

**DOI:** 10.1002/cbic.70464

**Published:** 2026-07-14

**Authors:** Yichi Zhang, Ning Zhang, Linkai Jiang, Zhilong Zhou, Yuwei Zhang, Yuwen Wang, Yiting Lei, Ning Hu, Zhong Alan Li

**Affiliations:** ^1^ Department of Biomedical Engineering The Chinese University of Hong Kong NT, Hong Kong SAR China; ^2^ Department of Orthopedic Surgery The First Affiliated Hospital of Chongqing Medical University Chongqing China; ^3^ Department of Mechanical Engineering Tsinghua University Beijing China

**Keywords:** biomimetic scaffold, hydrogel, hydrogel microsphere, microfluidics, organ‐on‐a‐chip, regenerative medicine, tissue engineering

## Abstract

As an emerging microphysiological system, organ‐on‐a‐chip (OoC) replicates human organ structures and functions through microfabrication, holding promise as novel platforms for drug screening, disease modeling, and toxicity testing, although their broad application still faces significant validation challenges. This review systematically examines biomimetic scaffold selection strategies in OoC systems, with a focus on hydrogels, hydrogel microspheres, and other scaffold types. It details chip fabrication processes, including design concepts, material selection, microfabrication technology, and cell sources. By comparing physicochemical properties, biocompatibility, and functional characteristics of different materials, this paper explores optimal scaffold strategies for specific organ simulations. Based on comprehensive analysis, hydrogel scaffolds have become widely adopted for OoC construction due to their excellent biocompatibility and extracellular matrix‐like properties. Synthetic polymers and bioceramic scaffolds demonstrate significant value in meeting specific mechanical requirements and constructing complex microstructures. As a novel hydrogel form, hydrogel microspheres exhibit considerable potential in dynamic culture simulation and drug delivery owing to their high specific surface area and controllable release capabilities. This paper also discusses future directions, including personalized scaffold design, intelligent responsive materials, and multiorgan coupling systems, providing theoretical guidance for advancing OoC technology.

## Graphical abstract

Organ‐on‐a‐chip (OoC) serves as a promising microphysiological platform for drug screening, disease modeling, and toxicity assessment. Biomimetic scaffolds are core components that determine the performance of OoC systems by mimicking the in vivo extracellular microenvironment. This review systematically elaborates the selection, classification, fabrication, and application of diverse scaffolds including hydrogels, hydrogel microspheres, synthetic polymers, and bioceramics in OoC. Hydrogels stand out owing to superior biocompatibility and ECM‐mimicking properties, while hydrogel microspheres exhibit unique advantages in dynamic cell culture and controlled drug delivery. We further summarize targeted scaffold design strategies for typical organ chips such as bone‐joint, liver, heart, blood vessel, and skin, discuss current challenges including standardization, long‐term stability, and vascularization, and prospect future directions covering personalized design, intelligent responsive materials, and multiorgan coupling systems, aiming to provide theoretical guidance for the advancement of OoC technology.

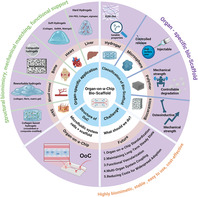

## 1 Introduction

Organ‐on‐a‐chip (OoC) can mimic the key structural and functional features of human organs at the microscale [1, 2]. This technology breaks through the limitations of traditional two‐dimensional cell culture, better reproducing the in vivo microenvironment and providing a more reliable platform for drug development, disease modeling, and toxicity testing [3, 4]. The core components of the OoC include microfluidic channels, cell culture chambers, and biomimetic scaffolds [5, 6]. Among them, the biomimetic scaffold, as a supporting structure for cell attachment, growth, and differentiation, not only provides physical support but also regulates cell behavior through its biochemical and mechanical properties, mimicking the extracellular matrix (ECM) environment in vivo [7, 8].

In OoC, the choice of biocompatible scaffolds directly affects the performance and reliability of the chip [9]. An ideal scaffold should possess good biocompatibility, an appropriate degradation rate, mechanical properties similar to natural tissues, and adjustable pore structures and surface characteristics [10]. The main types of biocompatible scaffolds currently used in OoC include hydrogel scaffolds, hydrogel microsphere (HM) scaffolds, synthetic polymer scaffolds, bioceramic scaffolds, decellularized ECM (dECM), and composite scaffolds [5, 11, 12, 13]. Each type of scaffold has its unique characteristics and applicable scenarios and needs to be selected according to the specific requirements of the simulated organ.

**FIGURE 1:**
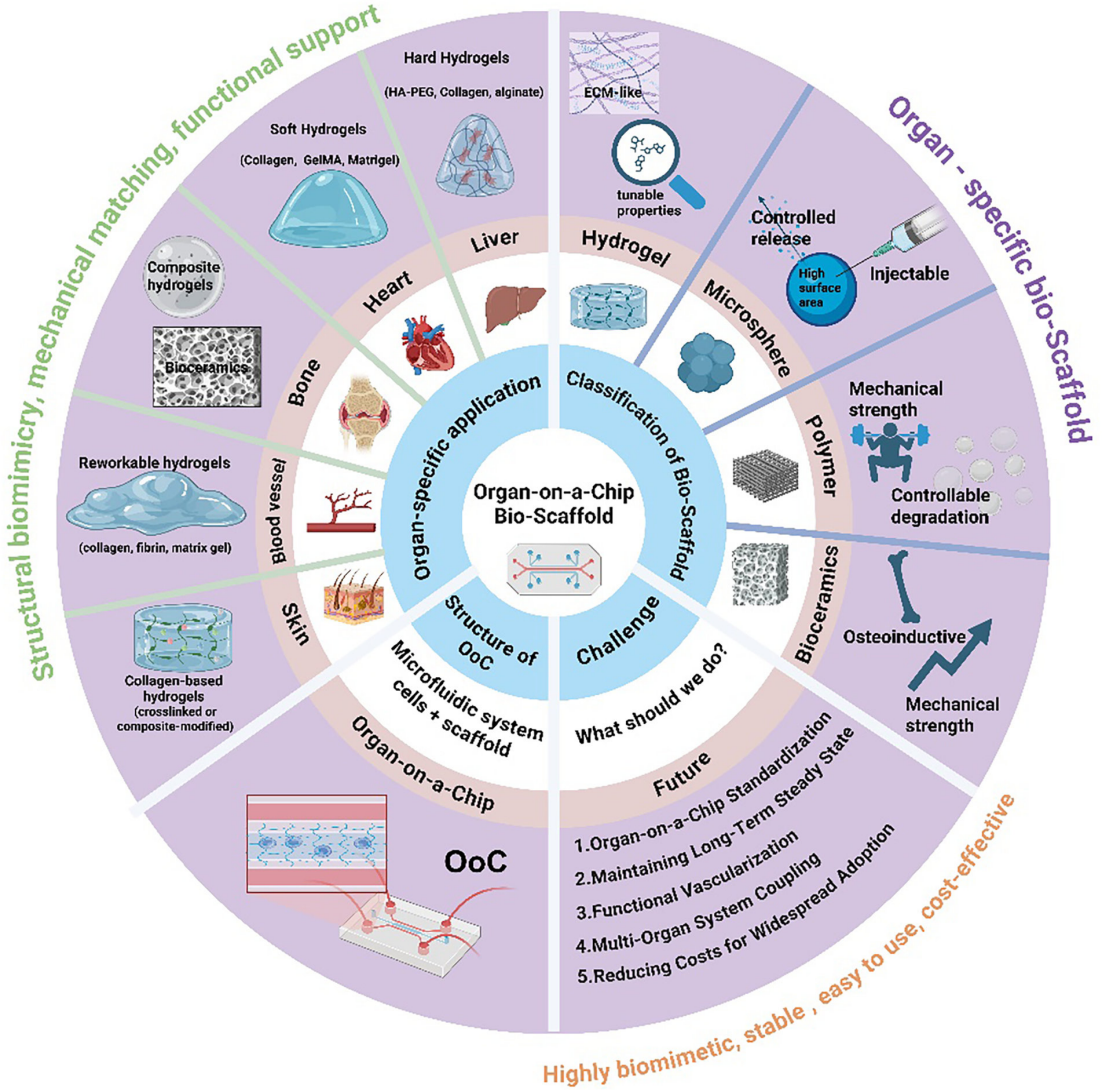
Overview of biomaterial scaffolds for OoC systems.

This article aims to comprehensively review the selection and application strategies of scaffolds in OoC, systematically analyze the advantages, limitations, and applicable conditions of different types of scaffolds, and provide guidance for the design and construction of OoC (Figure 1). The article will discuss in detail the fabrication methods of OoC, including design concepts, material selection, and processing techniques; deeply analyze the characteristics and applications of hydrogel scaffolds, HM scaffolds, and other types of scaffolds; summarize the application strategies for selecting biocompatible scaffolds for OoC of different organs; and look ahead to future development directions and challenges. Through this review, we hope to provide comprehensive reference and inspiration for scientific researchers engaged in the research and application of OoC.

## 2 Overview of OoC Technology

As an innovative product of the third‐generation in vitro model technology following traditional two‐dimensional planar cell culture and three‐dimensional cell culture models, OoC integrates cutting‐edge interdisciplinary breakthroughs in their development approach [14]. OoC is an innovative technology platform developed based on microfluidics and cell biology principles, aiming to simulate the structural and functional characteristics of human organs on a microchip [15, 16, 17, 18]. Such chips are usually made of transparent polymer materials (such as polydimethylsiloxane, PDMS), containing micrometer‐scale fluid channels and porous membranes inside [19, 20]. Living cells are seeded in the channels, and by precisely controlling fluid flow, mechanical stimulation, and biochemical factor gradients, the microphysiological environment of human organs is reproduced [21]. The core goal of OoC technology is to provide a more physiologically relevant in vitro model to bridge the translational gap between traditional two‐dimensional cell culture and animal experiments [22, 23].

The development of OoC stems from the crossintegration of multiple disciplines, including microfluidics, tissue engineering, materials science, and cell biology [24, 25]. As early as the beginning of the 21st century, researchers began to explore the use of microfabrication techniques to construct microfluidic devices for cell culture [26]. In 2010, the Wyss Institute at Harvard University first proposed the concept of “lung‐on‐a‐chip,” which can simulate the functions and mechanical breathing movements of alveoli in a microdevice. This groundbreaking work has triggered a global boom in the development of various OoCs [27]. To date, scientists have successfully developed a variety of organ models, such as lung, heart, liver, kidney, intestine, and brain chips [14, 27, 28, 29, 30, 31, 32, 33] and have begun to attempt to connect multiple OoCs to build a “human‐on‐a‐chip” system for evaluating the systemic effects of drugs in the body.

The importance of OoC is mainly reflected in the following aspects. First, it offers a more physiologically relevant in vitro model that has the potential to improve the prediction of drug responses and toxicity in humans and may ultimately contribute to reducing the failure rate of drug development [34]. However, to date, only a few OoC systems (e.g., the Emulate liver chip for DILI) have received regulatory qualification, highlighting the substantial translational and validation hurdles that remain. Second, OoC technology can reduce the dependence on animal experiments and conform to the 3R (Replacement, Reduction, Refinement) principle. In addition, OoC provides a powerful tool for personalized medicine. Disease‐specific models can be constructed using patient‐derived cells to achieve personalized drug screening and treatment optimization.

A typical OoC system usually consists of three key functional modules: a microfluidic channel network, a bioscaffold, and a cell culture chamber. Among them, the microfluidic channels are responsible for achieving the directional perfusion of the culture medium, the excretion of metabolic wastes, and the transmission of mechanical stimuli; the bioscaffold provides a bionic three‐dimensional growth environment for cells to support their adhesion, proliferation, and differentiation, while the cell culture chamber, as the core functional unit of the chip, is the physical space that bears the bioscaffold and cells and realizes the construction of a tissue‐specific microenvironment. Its structure and design directly determine the spatial arrangement of cells, cell–cell interactions, and the final expression of tissue functions. These three parts cooperate closely to jointly simulate the complex microenvironment of organs in the body. Next, we will discuss the design and fabrication process of OoC in detail.

## 3 Design and Fabrication of OoC

### 3.1 Design Concepts and Principles

The design of OoC needs to comprehensively consider various factors such as biological simulation accuracy, manufacturing feasibility, and experimental operability. The core design concept is to achieve the simulation of the key functions of the target organ with a minimized complex structure. In the design process, engineers need to cooperate closely with biologists and clinicians to determine the key characteristics and functional indicators of the organ to be simulated.

In the design of OoC, the following core principles are usually followed to ensure its effective simulation of the human physiological environment: Function‐oriented principle ‐ focusing on replicating the core physiological or pathological functions of the target organ rather than its complete anatomical structure; structural bionics and scale matching principle ‐ simulating the key structural features of tissues (such as barriers, lumens) at the microscale and making the physical dimensions of the chip (such as channel width, chamber height) match the physiological scale of cell and tissue functional units; dynamic perfusion principle‐simulating blood or body fluid flow through controlled fluid circulation to achieve nutrient delivery, waste removal, and the application of mechanical stimuli (such as shear stress); real‐time monitoring and interface convenience principle ‐ integrating sensing modules or reserving optical and electrical interfaces for easy real‐time monitoring of cell activities and nondestructive sampling; modularity and integrability principle adopting a standardized design to support the interconnection of multiorgan modules and system expansion to construct more complex multiorgan coupling systems [35, 36].

### 3.2 Material Selection

The selection of materials for OoC fabrication has an important impact on its performance and applications. Ideal chip materials should possess biocompatibility, optical transparency, gas permeability, processability, and cost‐effectiveness. The most commonly used material at present is PDMS, which has excellent optical transparency, gas permeability, and elasticity, facilitating microfabrication [37]. However, PDMS also has some limitations. For example, the protein adsorption problem caused by hydrophobicity requires oxygen plasma treatment or modification with a polyethylene glycol (PEG) coating to enhance hydrophilicity [38]. Other commonly used materials include polymethyl methacrylate (PMMA), polycarbonate (PC), and cycloolefin copolymer (COC). In recent years, some new materials have emerged, such as temperature‐responsive polymers, hydrogel integrated materials, etc., providing more functional possibilities for OoC [39, 40, 41, 42].

### 3.3 Microfabrication Techniques

Microfabrication techniques are the core processes for constructing OoC. Currently, they mainly include methods such as soft lithography, micromilling, hot embossing, and 3D printing (Table 1). Among them, soft lithography and 3D printing have become the two most representative and widely used technologies in this field due to their significant advantages in terms of structural flexibility, material compatibility, and operation convenience [48].

**TABLE 1:** Comparison of main microfabrication technologies for OoC.

Technology type	Advantages	Limitations	Applicable materials	References
Soft lithography	High resolution, low cost, easy to operate	Complex master mold fabrication, difficult for mass production	PDMS	[5, 43, 44]
3D printing	High design freedom, rapid prototyping	Limited resolution, material limitations	Photosensitive resin, special bioink	[6, 45]
Hot embossing	Suitable for mass production, low cost	High‐temperature and high‐pressure conditions, low design flexibility	Thermoplastic polymer	[46]
Micro‐milling	Direct machining, no need for master mold	Limited resolution, rough surface	PMMA, PC	[47]

**FIGURE 2:**
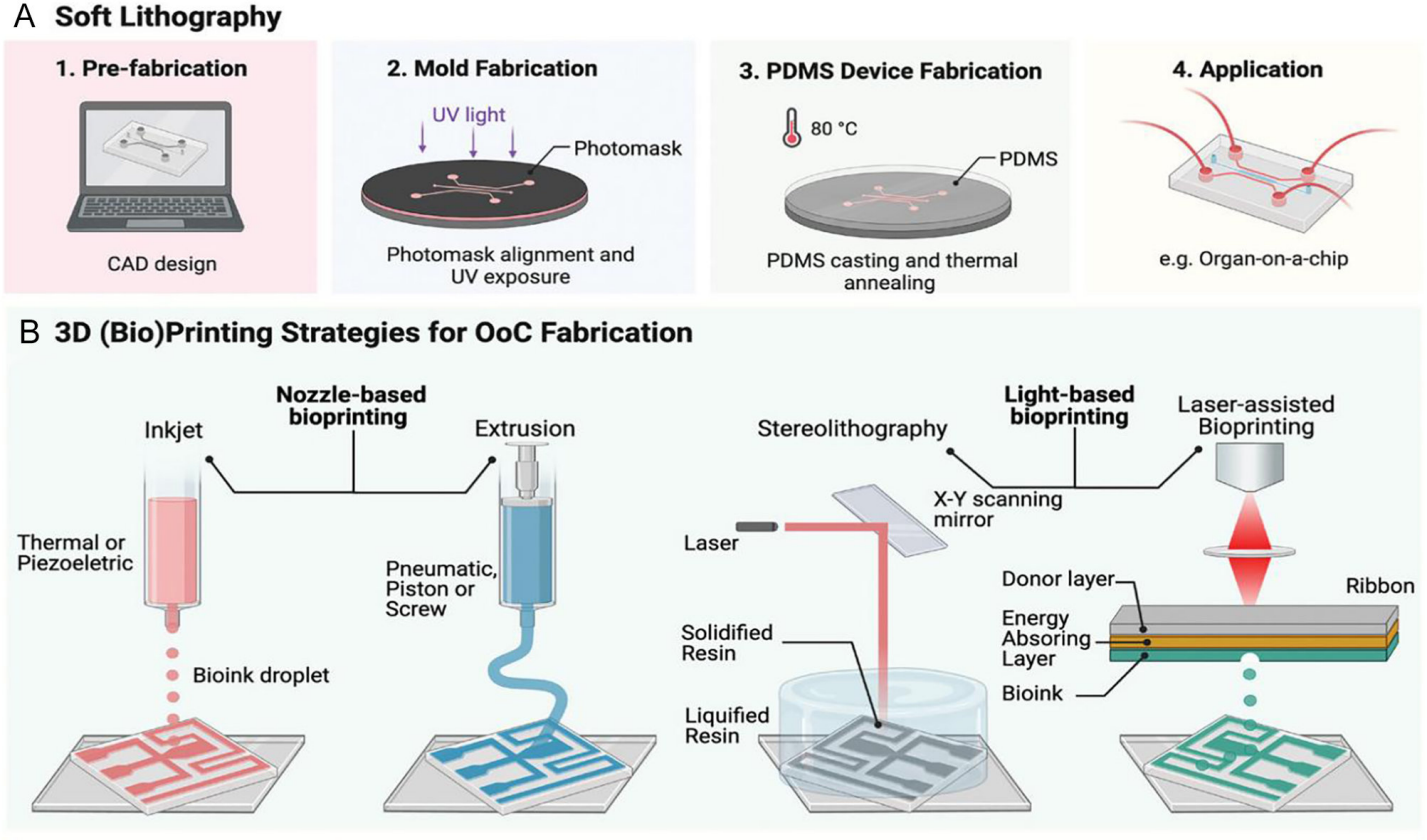
Representative methods for OoC fabrication. (A) Typical steps for fabricating OoC through soft lithography, including pattern design, mask and master fabrication, replica molding, and the generation of OoC. (B) Schematic diagram of 3D bioprinting strategies for engineering OoC, including nozzle‐based inkjet and extrusion techniques, as well as light‐based SLA and laser‐assisted techniques. Adapted with permission [50]. Copyright 2025, Wiley‐VCH.

Soft lithography is the most commonly used method at present. Soft lithography has characteristics such as high resolution, versatility, rapid prototyping ability, low cost, and integrability and is widely used to construct complex microscale architectures, physiological barriers, and multilayer microchannel systems. The standard soft lithography workflow usually involves several steps: photomask design and fabrication, substrate preparation, photoresist spin coating, soft baking, ultraviolet irradiation, development, and cleaning, followed by postlithography processes such as etching or material deposition [44, 49]. In OoC fabrication, soft lithography typically uses negative photoresists such as SU‐8 to create high‐resolution master molds on silicon wafers. Then, the PDMS prepolymer is cast on the mold, cured, and peeled off to produce PDMS replicas with well‐defined microstructures. Subsequently, these PDMS layers are usually bonded to glass or other PDMS layers through oxygen plasma treatment to form a closed microfluidic system (Figure 2A). Despite some limitations, such as the absorption of hydrophobic small molecules by PDMS and the challenge of applying this method to rigid materials, the flexibility, precision, and rapid prototyping ability of soft lithography make it the most practical and widely used microfabrication method in current OoC development [5].

Compared with soft lithography, 3D bioprinting has several advantages in the medical and biomedical fields, such as shorter turnaround times and a wider range of material choices [48, 51]. This method requires extremely low microfabrication expertise, allowing the simultaneous or sequential printing of polymers, hydrogels, and multiple cell types, thus enabling the fabrication of customized, repeatable, and complex patient‐specific three‐dimensional biomimetic tissue constructs and achieving precise cell positioning [52].

As an important means of constructing complex biological structures, 3D bioprinting technology can be mainly divided into nozzle bioprinting (including inkjet and extrusion) and light‐based bioprinting (such as stereolithography [SLA] and laser‐assisted printing) (Figure 2B) [53]. In addition to device manufacturing, 3D printing technology is also applied to the preparation of tissues or scaffolds for OoC models. Laser‐assisted printing technology is particularly prominent, achieving high droplet resolution through precise laser positioning. In short, 3D printing technology has brought significant potential for promoting the development of OoC. This technology not only provides diverse processing methods in the field of microfluidic chip manufacturing but also significantly improves manufacturing precision, thus strongly promoting the research and application of OoC for basic construction. 3D printing technology has important potential in constructing skeletal muscle chips, but its practical application still faces key limitations, including insufficient printing resolution to achieve cell‐level biomimetic arrangement, insufficient biocompatibility, and mechanical properties of existing biomaterials, and the constructed models are still relatively limited in simulating dynamic physiological functions such as muscle contraction, metabolism, and cross–tissue interaction [6].

### 3.4 Cell Sources

The cell sources of OoC are key factors determining its physiological relevance, functional stability, and research value. Common cell sources include primary cells, cell lines, mesenchymal stem cells (MSCs), and induced pluripotent stem cells. Primary cells are directly derived from the body and retain the natural physiological characteristics and functions of in vivo cells, thus directly reflecting the functional characteristics of tissues. However, due to individual differences in their sources, their proliferative capacity is poor, and organ models have significant heterogeneity [54]. Cell lines are immortalized cells that overcome the limitations of the proliferative capacity of primary cells and provide high stability and convenience for long‐term research. Despite the well‐known limitations of genetic drift and altered phenotypes, immortalized cell lines offer unique advantages for OoC development: (i) exceptional batch‐to‐batch consistency, (ii) indefinite proliferative capacity enabling large‐scale experiments, and (iii) decades of historical data on their behavior, drug responses, and genetic characteristics. These features make them invaluable for high‐throughput drug screening and assay standardization, particularly in early‐stage compound testing where reproducibility is paramount. However, researchers should be aware that immortalization and extended culture time may alter natural cell characteristics, and some cell lines lose specific in vivo functions. The choice between primary cells, cell lines, or induced pluripotent stem cells (iPSC)‐derived cells should be guided by the specific research question and the trade‐off between physiological relevance and experimental practicality [55]. MSCs are one of the most commonly used cell sources for organ models and have the potential to differentiate into multiple cell types. They also have significant immunosuppressive and regulatory capabilities, making them a focus of cell therapy. MSCs can be extracted from various tissues such as bone marrow, adipose tissue, umbilical cord, and dental pulp. However, MSCs are sensitive to the in vitro environment, and when using MSCs as the cell source for organ models, strict control of the extracellular microenvironment is required [56, 57]. iPSCs are pluripotent stem cells obtained by reprogramming somatic cells and have the potential for both unlimited proliferation and differentiation into various functional cells. Their core advantage is the ability to construct personalized and disease‐specific organ models, providing an ideal platform for precision medicine and drug testing. However, the functional maturity of cells differentiated from iPSCs may be insufficient, and there are challenges such as complex culture processes, high costs, and residual pluripotency. Nevertheless, it remains a key driver for promoting the clinical translation of OoC and high‐fidelity simulation of human physiology [58].

## 4 Biomimetic Scaffolds in OoC

**FIGURE 3:**
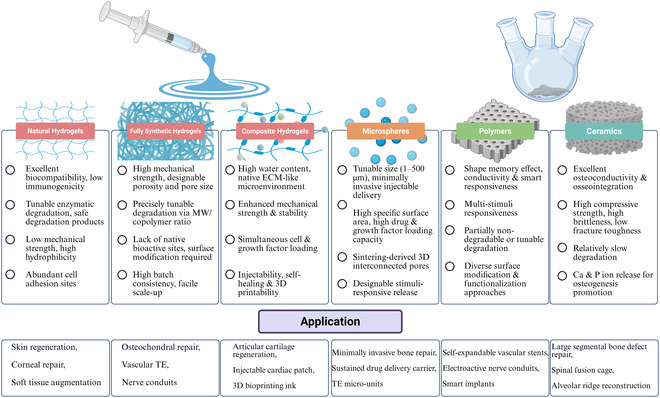
A comparative summary of six biomaterial scaffold types—natural hydrogels, fully synthetic hydrogels, composite hydrogels, microspheres, polymers, and ceramics—detailing their key properties (biocompatibility, mechanical strength, degradability, bioactivity) and representative applications.

In an OoC system, as a physical carrier for cell attachment, growth, and functional expression, the selection and design of the bioscaffold directly affect the physiological relevance and functional reliability of the chip. This section systematically reviews the material properties, preparation strategies, and application advantages of various bioscaffolds in OoC, focusing on discussions around the main types such as hydrogel scaffolds, HMs, synthetic polymers, and bioceramics (Table 2). By analyzing the physicochemical properties, biocompatibility, structural controllability of different scaffolds, and their applicability in simulating specific tissue microenvironments, this section aims to provide a theoretical basis and technical reference for the scaffold selection of OoC and promote the construction of more physiologically realistic in vitro models with improved physiological authenticity (Figure 3).

**TABLE 2:** Types, materials, characteristics, and applications of commonly used biomaterials in OoC.

Biological scaffold type	Specific materials	Main characteristics and applications	References
Hydrogel scaffolds	Natural hydrogels: Collagen, gelatin, hyaluronic acid, alginate, chitosan	High water content, similar to natural ECM, good biocompatibility, suitable for soft tissue chips such as liver, kidney, and brain	[29, 30, 33, 34, 35, 39, 40, 41, 42, 48]
	Synthetic hydrogels: PEG, polyacrylate (such as PLGA, PCL)	Regulable mechanical properties, controllable degradation, easy to functionalize, suitable for high‐precision structural requirements	[6, 30, 43, 44, 45, 46]
HMs	Microspheres prepared from materials such as alginate, gelatin, and chitosan	Large specific surface area, can be dynamically filled, support cell encapsulation and controlled drug release, suitable for dynamic culture and drug delivery	[50, 52, 53, 54, 55, 56, 57, 58, 59, 60, 61]
Synthetic polymer scaffolds	PLA, PGA, PCL, PIC	Good mechanical properties, adjustable degradation rate, suitable for chips that require mechanical support such as bone and heart	[62, 63, 64, 65, 66, 67, 68, 69, 70]
Bioceramic scaffolds	Hydroxyapatite (HA), β‐tricalcium phosphate (β‐TCP)	Strong osteoconductivity and osteoinductivity, suitable for bone chips and bone tissue simulation	[71, 72]

### 4.1 Hydrogel Scaffolds

Hydrogel block scaffolds are polymeric materials composed of hydrophilic polymer networks that can absorb and retain large amounts of water without dissolving [59]. Their unique physicochemical properties make them one of the most ideal biomaterial scaffolds for OoC applications.

#### 4.1.1 Characteristics and Advantages of Hydrogel Scaffolds

Hydrogel scaffolds have become key materials in the fields of tissue engineering and OoC due to their comprehensive advantages of a series of biomimetic, adjustable, and functionalized properties. These characteristics enable them to highly mimic the microenvironment of natural ECM, thus effectively supporting cell survival, functional expression, and tissue construction. Specifically, their core advantages are mainly reflected in the following aspects.

##### 4.1.1.1 Three‐Dimensional Environment With High Water Content

Hydrogels usually contain 80%–99% water, which is very close to the highly hydrated nature of natural ECM and can provide a hydrophilic microenvironment for cells, facilitating nutrient diffusion and waste excretion [60].

Adjustable physical and chemical properties: By changing the type of polymer chains, crosslinking density, and adding functional groups, the mechanical properties (such as elastic modulus, elastic modulus), degradation rate, and pore structure of hydrogels can be precisely regulated to match the characteristics of specific tissues [61].

##### 4.1.1.2 Excellent Biocompatibility

Many hydrogel materials (especially those of natural origin) have good biocompatibility, support cell adhesion, proliferation and differentiation, and usually do not cause obvious immune responses [73].

##### 4.1.1.3 Surface Functionalization Ability

The surface of hydrogels can be functionalized by introducing bioactive molecules (such as RGD peptides, growth factors, enzyme‐sensitive sites), thereby regulating cell behavior and tissue formation [74].

##### 4.1.1.4 Injectability and In‐Situ Forming

Some hydrogels have temperature or pH‐responsive properties, can be mixed with cells in a solution state and then injected into the target site, and then form a gel under physiological conditions, achieving minimally invasive implantation and close fitting with the defect site [75]. Throughout this review, we primarily use the term elastic modulus to describe mechanical properties, unless the cited work specifically uses other metrics.

#### 4.1.2 Classification of Hydrogel Materials

When selecting hydrogel materials suitable for OoC, multiple key factors need to be considered comprehensively, including the type of target organ, the required mechanical properties, the material degradation characteristics, and its bioactivity requirements. Hydrogels can generally be divided into natural sources (such as gelatin) and synthetic categories (such as polycaprolactone). The following will outline the commonly used hydrogel materials in the field of OoC and their main characteristics.

Natural polymer hydrogels are derived from natural resources and are mainly extracted from animal tissues (such as collagen), plant tissues (such as cellulose), and microbial metabolites (such as exopolysaccharides) [76]. Such materials usually possess excellent biocompatibility, good cell adhesion, biodegradability, and low toxicity and are thus regarded as ideal hydrogel matrix components in a variety of biomedical applications [77]. In the field of OoC, commonly used natural‐derived hydrogels mainly include protein‐based materials (such as collagen and gelatin) and polysaccharide‐based materials (such as chitosan, alginate, and hyaluronic acid).

### 4.2 Natural Hydrogel Materials

• Collagen: It is the main component of the animal ECM, composed of three α‐chains that form a triple helix structure, accounting for 25%–30% of the total protein in mammals [78, 79]. It exhibits good cytocompatibility and biodegradability, and its pore size and elastic modulus can be tuned by adjusting the concentration [62]. As a natural ECM component, collagen promotes cell adhesion and proliferation, making it widely used in fields such as skin regeneration, bone tissue engineering, and 3D bioprinting [63]. However, collagen is mainly extracted from animal tissues, and its xenogeneic origin may trigger innate immune responses in vitro, which limit its application in long‐term toxicity assessment assays.
• Gelatin: Gelatin is a biocompatible protein derived from the hydrolysis of collagen and is one of the most widely used natural polymers due to its biodegradability, biocompatibility, biosafety, and cost‐effectiveness [64, 65]. The collagen part of gelatin is hydrolyzed, retaining the RGD cell adhesion sequence, and its price is lower than that of collagen. However, gelatin has limitations such as low mechanical stability, rapid enzymatic degradation in vivo, and large batch‐to‐batch variations [66]. To address these issues, researchers have mixed gelatin with polymers such as hyaluronic acid and alginate to improve adhesion, shear‐thinning behavior, and stability [67]. Similar to collagen, gelatin derived from animal sources also has potential immunogenicity risks.
• Hyaluronic acid: Hyaluronic acid (HA) is a glycosaminoglycan composed of repeating units of N‐acetylglucosamine and glucuronic acid and is widely present in synovial fluid, skin, and other tissues [68]. It has good lubricity and cell signal regulation functions, and its mechanical properties can be improved through chemical modification.
• Alginate: Alginate is a natural polysaccharide extracted from brown algae, consisting of an alternating polymer of β‐D‐mannuronic acid (M unit) and α‐L‐guluronic acid (G unit). Naturally sourced alginate is nontoxic, nonimmunogenic, and suitable for in vivo applications (e.g., drug delivery, tissue engineering) [69].
• Chitosan: Chitosan is a cationic polysaccharide obtained from the deacetylation of chitin, mainly sourced from crustacean shells [70]. Its broad‐spectrum antibacterial properties make it suitable for colon‐targeted drug delivery systems, antibacterial dressings, and stem cell engineering [71]. However, chitosan also has limitations, such as rapid degradation in neutral environments, solubility limited to weakly acidic media, and low elastic modulus in its pure form [72].

A critical limitation of naturally derived hydrogels is their intrinsic immunogenicity. Xenogeneic components (e.g., animal collagen, Matrigel, gelatin) can elicit inflammatory responses in coculture systems containing immune cells or when used for in vivo transplantation. This immune activation may confound the interpretation of drug toxicity and efficacy assays, particularly for immunomodulatory compounds. Researchers should therefore consider the immunogenicity of natural hydrogels when designing OoC models and where possible, use species‐matched or fully synthetic alternatives to minimize background immune effects.

Due to limitations such as insufficient mechanical properties often associated with natural polymers, organic chemical synthesis strategies have been frequently employed in recent years to improve their performance. By screening or modifying basic building units, the physicochemical properties and mechanical behavior of hydrogels can be systematically regulated [77]. Additionally, hydrogels based on pure synthetic polymers are also widely used in the construction of OoC platforms. Among them, PEG is one of the most commonly used synthetic polymers, and its flexible chemical structure allows for various functional modifications to meet different biocompatibility and mechanical design requirements [80]. Another synthetic material commonly used in high‐quality manufacturing (such as the construction of high‐precision OoC) is polyacrylate. This material can flexibly control its mechanical elastic modulus by adjusting parameters such as crosslinking density, enabling its elastic modulus to be adjustable within a wide range from less than to several megapascals, thus adapting to the mechanical microenvironment requirements of different tissue types [81].

### 4.3 Synthetic Hydrogel Materials

• Polyethylene glycol (PEG): PEG is regarded as a “gold standard” synthetic polymer with excellent biocompatibility and high customizability. Through physical adsorption or chemical modification, PEG can significantly extend the half‐life of therapeutic molecules and enhance their efficacy [82]. This polymer can be modified to produce various derivatives, such as diacrylate, dithiol, or diacrylamide, etc [83]. These modifications not only enable the hydrogel to load abundant bioactive molecules (such as polypeptides) [84], but also enhance the reactivity and adhesion properties of the gel, thus being better applicable to advanced manufacturing technologies such as 3D printing [85, 86]. A critical design consideration for synthetic hydrogels in OoC is their swelling behavior. Hydrogels absorb water and expand, which can alter the dimensions of microfluidic channels, change pore structures, and affect mechanical properties. Swelling ratios vary widely (e.g., PEG hydrogels can swell by 10%–200% depending on crosslinking density). Researchers should characterize and control swelling to prevent clogging or delamination within OoC devices.
• Polyacrylates (such as PLGA, PCLA): Among polyacrylate materials, poly(lactic‐co‐glycolic acid) (PLGA), as a typical biodegradable aliphatic polyester, has adjustable monomer ratios and molecular weights, so its degradation rate and mechanical properties can be precisely regulated. However, the acidic products generated during its degradation may lead to a decrease in the local microenvironment pH, and this characteristic needs to be noted in some applications [87].
• Polyvinyl alcohol: Polyvinyl alcohol (PVA) is a water‐soluble synthetic polymer formed by the hydrolysis of polyvinyl acetate, characterized by a high hydroxyl content and adjustable crystallinity. It has high elastic modulus, biodegradability, excellent biocompatibility, as well as nontoxic and nonirritating properties. PVA is usually used to prepare hydrogels for disease treatment [88, 89].
• Polyisocyanide (PIC) hydrogels: PIC is a fully synthetic, thermosensitive hydrogel that undergoes a sol–gel transition at physiological temperature. Unlike conventional static hydrogels, PIC exhibits unique reversible liquefaction upon cooling and shear‐thinning injectability. These properties enable its use as a removable dynamic scaffold for OoC applications – for example, cells can be encapsulated in a gel at 37 °C, and the scaffold can be liquefied on demand by mild cooling to retrieve cells or study matrix remodeling without enzymatic digestion. PIC hydrogels also support the formation of injectable microtissues, offering new possibilities for modular OoC construction. Despite these advantages, the application of PIC in OoC is still emerging and warrants further investigation.

#### 4.3.1 Challenges and Improvement Strategies of Hydrogel Scaffolds

Although hydrogels have many advantages, they still face some challenges in practical applications:

##### 4.3.1.1 Insufficient Elastic Modulus

Many hydrogels (especially natural hydrogels) have low elastic modulus and are difficult to maintain structural integrity in the long term. Solutions include developing composite hydrogels (such as nanocellulose‐reinforced hydrogels), double‐network hydrogels, and special crosslinking strategies.

##### 4.3.1.2 Degradation Rate Control

The mismatch between the hydrogel degradation rate and the tissue regeneration rate will affect the long‐term performance of the chip. By adjusting the crosslinking density, using enzyme‐sensitive crosslinkers, or compounding materials with different degradation rates, more precise degradation control can be achieved.

##### 4.3.1.3 Bioactivity Regulation

Pure hydrogels may lack necessary bioactive signals. The solution is to introduce bioactive molecules (such as growth factors, cytokines) or cell adhesion peptide segments (such as RGD, YIGSR) to create biofunctionalized hydrogels.

##### 4.3.1.4 Manufacturing Precision Limitation

Hydrogel scaffolds fabricated by traditional methods often have limited structural precision. Combining 3D printing (such as digital light processing‐based photocurable bioprinting) can fabricate high‐precision hydrogel scaffolds with complex microstructures.

Among the emerging strategies, dynamic‐responsive hydrogels have attracted growing interest. These materials can respond to biological cues (e.g., enzymes, pH, redox state) or external stimuli (e.g., light, magnetic field, temperature) to achieve spatiotemporal control over gelation, degradation, or mechanical properties. For example, thermoreversible polyisocyanide (PIC) hydrogels enable reversible sol–gel transition, allowing nonenzymatic cell retrieval and injectable microtissue formation. Incorporating such dynamic responsiveness into OoC scaffolds will allow more precise recapitulation of physiological processes such as matrix remodeling, cell migration, and drug‐triggered release.

### 4.4 Hydrogel Microspheres

HMs are spherical gel particles with sizes ranging from micrometers to millimeters. Featuring a large specific surface area and tunable pore architecture, they stand out as promising scaffold materials for OoC (OoC) systems. This section focuses specifically on HMs, a key research topic in our group, since their potential for OoC applications remains underexplored in existing literature. We herein systematically discuss their fabrication, merits, and practical applications. Compared with bulk hydrogels, microspheres offer a larger specific surface area to support cell adhesion and mass transport. As particulate materials, they can dynamically fill irregular cavities and self‐assemble into porous frameworks. Additionally, HMs can act as carriers for bioactive factors, enabling sustained and controlled factor release.

#### 4.4.1 Preparation Methods of Hydrogel Microspheres

HMs can be prepared by a variety of techniques. Commonly used methods include emulsion crosslinking, microfluidics, electrostatic droplet injection, aerodynamic microfluidics, etc. Each method has its own characteristics in terms of microsphere size regulation, targeting specificity, drug‐loading capacity, and degradation behavior. In practical applications, a comprehensive selection needs to be made according to specific requirements [90]. The hydrogel microparticles prepared by these techniques are highly promising biomedical carriers, and their further integration with emerging materials and OoC systems represents a broad development direction in the field of biomedical engineering [66].

##### 4.4.1.1 Emulsion Crosslinking Method

Batch emulsification is a technique that disperses an aqueous solution into an oil phase to form emulsion droplets through mechanical stirring or homogenization and then obtains microspheres through chemical crosslinking or physical curing [91]. This method has simple equipment, low cost, and is easy to scale up, with high cost‐effectiveness and scalability, suitable for large‐scale production. However, the microspheres generated by this method often have a relatively wide size distribution, and the control precision of the shape and size of the microspheres is relatively limited [92]. In the biomedical field, this technique is commonly used to prepare drug‐loaded HMs to achieve controllable drug release [93].

##### 4.4.1.2 Microfluidic Method

Using a microfluidic device, monodisperse droplets can be generated by precisely controlling the fluid flow in microchannels and then cured into microspheres with uniform size through photopolymerization or ionic crosslinking [94]. This technology has good precision and designability, can achieve precise control of the size and morphology of microspheres, and is suitable for preparing biomedical microparticles with high monodispersity and meeting strict requirements [95]. Therefore, microfluidic technology has been widely applied in the fields of drug delivery, tissue engineering, and biosensors. However, this technology also faces limitations such as high equipment cost, relatively complex operation, and limited production efficiency, which need to be comprehensively considered in actual large‐scale applications [96].

##### 4.4.1.3 Electrostatic Dripping Method

The electrohydrodynamic spraying technology uses a high‐voltage electric field to atomize the polymer solution into charged fine droplets, and then the droplets are cured by solvent evaporation to obtain microspheres in the nano‐ to microscale. This method is particularly suitable for preparing drug‐loaded microspheres using synthetic materials (such as PLGA) [97]. Although this technology can obtain microspheres with relatively uniform sizes, its process depends on high‐voltage equipment, the operating conditions are relatively strict, and the high‐voltage environment may affect the structure and function of some bioactive substances [98].

##### 4.4.1.4 Aerodynamic Microfluidics

The pneumatic microfluidic technology uses the shear force and kinetic effects induced by air flow to disperse the precursor solution into microdroplets with uniform size and precisely controls the formation and curing process to prepare HMs with good monodispersity. By adjusting key parameters such as nozzle diameter and air flow velocity, flexible control of the microsphere size can be achieved. This method has good scalability, can efficiently prepare a large number of monodisperse microspheres in a short time, and is suitable for large‐scale production requirements [99].

#### 4.4.2 Advantages and Applications of Hydrogel Microspheres

As a commonly used scaffold material for three‐dimensional cell culture, HMs have received extensive attention in OoC technology due to their excellent biocompatibility, tunable physicochemical properties, and unique microscale structure.

### 4.5 Provide a Biomimetic Three‐Dimensional Culture Microenvironment

Compared with the traditional two‐dimensional culture mode, the three‐dimensional culture system based on HMs can more realistically simulate the in vivo cell microenvironment, provide a support structure closer to the physiological state for cells, and thus significantly improve the biofidelity and functional performance of the OoC system. For example, Tian et al. designed a novel PDMS‐based microfluidic platform with a T‐shaped structure improved by a molding process, which can be used to prepare HMs and microrods encapsulating live cells. By adjusting the flow rate ratio and channel constriction ratio, the diameter (300–1000 μm) and aspect ratio (1.3–3.6) of the generated microspheres can be conveniently controlled. Based on this technology, the researchers achieved the efficient preparation of highly uniform and shape‐predictable HMs and microrods. Fibroblasts and endothelial progenitor cells (ECFCs) encapsulated in microspheres and microrods with different diameters and aspect ratios all showed continuous normal cell behavior, and the loss of cell viability was negligible. While this study successfully demonstrates the feasibility of producing cell‐laden microspheres with controlled geometry, it does not provide an in‐depth characterization of the three‐dimensional microenvironment (e.g., matrix remodeling, nutrient gradient formation) nor does it establish a direct link between microsphere geometry and specific cellular functions. Thus, further studies are needed to validate the claimed biomimetic advantages. This work provides an important approach for the scalable production of large cell‐loaded microspheres with ideal geometric shapes, contributing to the promotion of their development in various biomedical applications [100].

### 4.6 Achieving Efficient Encapsulation, Protection, and Delivery of Cells

HMs can provide an ideal growth and protection microenvironment for cells, support the efficient encapsulation, targeted delivery, and high‐throughput screening of cells, and maintain a high cell viability and normal biological functions during long‐term culture. To optimize the cell encapsulation and subsequent screening processes, researchers are also continuously developing more gentle and efficient dynamic microarray platforms. Taking the novel system designed by Tan and Tsuchiya [101], as an example, this system integrates microfluidic technology and a bubble‐driven laser manipulation mechanism, which can achieve precise capture and release of microspheres encapsulating cells, and the operation process causes no obvious damage to the viability of the encapsulated cells. To alleviate the thermal effects that may be caused by laser operation, the research team effectively reduces the heat accumulation during bubble formation by introducing a nucleation chamber and using a low‐boiling‐point fluid, thus further improving the safety of the operation. Experimental results show that the cells encapsulated in alginate microspheres by this system still maintain a high viability, demonstrating its application prospects in high‐throughput cell screening and phenotypic analysis.

In addition to improvements in operational techniques, the long‐term biocompatibility and protective ability of HMs themselves have also attracted much attention. For example, Workman et al. successfully prepared monodisperse alginate microspheres through microfluidic technology and used them to encapsulate immortalized human cells [102]. During the 90‐day culture observation, the human embryonic kidney cells (HEK293) encapsulated in the microspheres still maintained a high survival rate, indicating that this type of microsphere system can provide a continuous and stable three‐dimensional microenvironment for internal cells and is suitable for research and application scenarios that require long‐term culture or dynamic monitoring.

### 4.7 Construction of Cell Niche and Complex System Models

HMs can be used to simulate the in vivo cell niche and construct a complex system model with multicellular interactions, providing a new platform for reproducing organ development processes, studying disease mechanisms, and conducting multiorgan interaction research. By encapsulating cells in HMs, these microcarriers can not only provide three‐dimensional structural support but also actively regulate the biochemical and mechanical signals in the surrounding microenvironment, thus effectively guiding cell behavior and coordinating cell–cell interactions. Recent studies have shown that HMs can be used to construct more complex and physiologically relevant three‐dimensional models, accurately simulating the cell niche and cell–cell communication in vitro and providing a highly biomimetic experimental platform for the study of disease mechanisms, drug responses, and tissue repair.

For example, Buonvino et al. designed an innovative three‐dimensional cell migration chip (3DCM‐Chip), which combines microfluidic technology with a “gel‐in‐gel” structure to study cell migration and invasion behaviors in a three‐dimensional environment [103]. By adjusting the physicochemical properties of the external gel and the arrangement of internal cells, this chip can precisely control the speed and mode of cell migration, thus simulating the complexity of the tumor microenvironment. This platform also supports the construction of coculture systems of multiple cell types, such as the coculture of tumor cells with fibroblasts or endothelial cells, helping to deeply analyze the impact of cell–cell interactions on tumor progression. Experimental results show that 3DCM‐Chip provides a highly controllable in vitro model for the study of the tumor microenvironment, capable of revealing the response mechanism of cells to biophysical and chemical stimuli in a biomimetic three‐dimensional environment, thus providing important experimental evidence for anticancer drug screening and treatment strategy optimization targeting the tumor microenvironment.

### 4.8 Support for Three‐Dimensional Network Structure and Mechanical Integrity

Due to their inherent pore structure and three‐dimensional network morphology, HMs have shown important value in the field of OoC, especially in constructing complex three‐dimensional tissue networks such as neural networks and vascular networks. For example, Tedesco et al. developed a three‐dimensional neural network scaffold based on chitosan microspheres [104]. These microspheres were prepared by physical cross‐linking method, with good hydrophilicity and adjustable mechanical properties. The research team successfully prepared chitosan microspheres with uniform size and water content higher than 98% using pneumatic atomization technology and cultured rat embryonic hippocampal neurons on them to construct a tightly structured, evenly distributed, and highly biomimetic three‐dimensional neuron network. Confocal microscopy and transmission electron microscopy observations showed that neurons not only formed a dense dendritic network on the surface of the microspheres, but their protrusions could also extend into the interior of the microspheres to form a more complex and interconnected three‐dimensional structure. In addition, electrophysiological analysis of this network using microelectrode arrays showed that the chitosan microsphere scaffold could support the long‐term culture and spontaneous discharge activities of neurons and exhibited good network synchrony. This study provides a stable and highly realistic three‐dimensional model basis for constructing functional brain chip systems and deeply exploring neural connections and network functions.

### 4.9 Facilitating Drug Screening and Drug Efficacy Testing

HMs can serve as drug carriers and diffusion models for drug screening, penetration, and clearance studies, providing a more physiologically relevant in vitro experimental tool for drug development. For example, Yu et al. developed a bilayer alginate core–shell microsphere platform based on microfluidics technology, which simplified the construction process of three‐dimensional tumor spheroids and could be used for efficient drug screening [105]. This platform used a two‐step flow focusing method to prepare microspheres with a uniform core–shell structure. Its core region was loaded with MCF‐7 cells (average core diameter of about 183 μm and shell thickness of about 70 μm), causing the cells to be mainly concentrated inside the spheroid, forming a spherical aggregate similar to a solid tumor. Compared with traditional single‐phase microspheres, this core–shell design could effectively prevent cell escape and adhesion to the culture medium bottom, thus extending the three‐dimensional culture period and ensuring the model purity. In the drug sensitivity test, the three‐dimensional spheroids in the core–shell microspheres showed higher half‐maximal inhibitory concentration (IC50) values for chemotherapeutic drugs (such as tamoxifen and docetaxel), reflecting their typical ability to mimic the multi‐drug resistance characteristics of solid tumors. This system avoided the step of frequently transferring microspheres in traditional methods, providing a simple, less interfering, and more physiologically relevant in vitro model for high‐throughput screening of anti‐cancer drugs.

### 4.10 Achieving Controlled Delivery and Release of Therapeutic Drugs

HMs have significant potential in the sustained delivery and controlled release of drugs and can serve as a stable and efficient carrier system for the development of precision therapy strategies. Carugo et al. designed a PMMA‐based biomimetic microfluidic platform to investigate the spatiotemporal release kinetics of doxorubicin‐loaded HMs under simulated vascular embolism conditions [106]. This platform constructed a microchannel network with a hierarchical branched structure to biomimetically simulate the complex microvascular system in tumor tissues and enabled precise injection and positioning of single drug‐loaded microspheres. It was found that by adjusting the Reynolds number (Re) and the restricted state of the microspheres in the channel (partially restricted or fully restricted), the spatial position and degree of constraint of the microspheres significantly affected the drug release rate and spatial distribution. Microspheres in a partially restricted state showed a higher drug release ratio, and the release behavior was nonlinearly related to the local flow rate, while fully restricted microspheres released more slowly and smoothly. The study further combined fluorescence imaging and spectral analysis to achieve high‐resolution spatial tracing of the drug release process, and the results showed that local flow field characteristics (such as vortex structures) could effectively promote drug diffusion behavior.

### 4.11 Synthetic Polymer Scaffolds

Synthetic polymer materials are another important type of scaffold materials in OoC. Compared with natural materials, they generally have better mechanical properties, adjustable degradation rates, and better consistency. Commonly used synthetic polymer materials include the following.

#### 4.11.1 Polylactic Acid (PLA)

Polylactic acid (PLA) is a biodegradable polyester, and its synthesis was recorded as early as 1780. Its production is usually achieved through chemical synthesis pathways, using acetaldehyde and carbohydrates for fermentation and polymerization reactions. The mechanical properties, biocompatibility, and degradation rate of this polymer are closely related to its molecular weight distribution, which can be regulated by means such as azeotropic dehydration condensation and extended polymerization time [107]. PLA is soluble in a variety of organic solvents, such as dioxane, tetrahydrofuran, and hot benzene. As a thermoplastic material, it can be processed into forms such as fibers or films, and its final mechanical properties depend on the scaffold manufacturing process used [108].

#### 4.11.2 Polyglycolic Acid (PGA)

Polyglycolic acid (PGA) is a linear aliphatic polyester with good biocompatibility and biodegradability, and it is nontoxic and does not trigger an immune response. It has been approved by the US Food and Drug Administration (FDA) for use in the biomedical field. PGA can be obtained from natural resources such as rice, wheat, and sweet potatoes through fermentation and polymerization processes [109], so it can be used as an alternative material to animal‐derived scaffolds. Such scaffolds have adjustable degradation rates and excellent processing properties, and thus are widely used [110]. PGA belongs to thermoplastic materials and can be flexibly formed and processed according to specific tissue engineering requirements [111]. Its degradation in vivo mainly occurs through nonenzymatic hydrolysis and is finally metabolized into nontoxic products.

#### 4.11.3 Polycaprolactone (PCL)

Polycaprolactone is a synthetic biodegradable polyester with a linear structure. Its preparation cost is relatively low, and it shows good processability and adaptability in scaffold design and manufacturing [112]. This aliphatic polymer has both suitable mechanical properties and biocompatibility and has been approved by the US Food and Drug Administration (FDA) and is widely used in the biomedical and tissue engineering fields. Due to its hydrophobic characteristics, the cell adhesion and biological interaction on its surface are usually weak [113]. For this reason, its surface is often functionalized by introducing bioactive amino acid sequences (such as RGD, GRGDSP, PSHRN, and IKVAV) or peptide segments (such as fibronectin fragments) [112]. In addition, PCL is often used as a copolymer component to enhance the overall stability of natural materials using its stable mechanical properties, while effectively improving the hydrophilicity, cell adhesion, and survival rate of the system [114].

### 4.12 Bioceramic Scaffolds

Bioceramic materials, especially calcium phosphate ceramics (such as hydroxyapatite HA, β‐tricalcium phosphate β‐TCP), have unique advantages in bone tissue simulation. They have excellent osteoconductivity and osteoinductivity and can promote osteoblast differentiation and bone matrix deposition, making them ideal scaffold materials for bone chips.

Atif et al. designed a microfluidic chip‐based platform that integrated hydroxyapatite into the cell culture chamber of the chip for dynamic culturing and evaluation of the growth, adhesion, and function of osteoblasts. Compared with traditional static culturing, microfluidic dynamic culturing provides fluid shear forces and nutrient delivery closer to those in vivo, enabling osteoblasts to exhibit more stable adhesion, growth, and mineralization behaviors on the surface of biomimetic ceramic materials [115]. Carter et al. further investigated the performance of highly reactive materials such as calcium‐deficient hydroxyapatite (CDHA) in the dynamic environment of the chip. The results showed that, compared with static culturing, dynamic culturing in the chip significantly increased cell activity and presented a potentially more anti‐inflammatory cell phenotype; after further loading the antioxidant drug Trolox into CDHA, the drug release and its effect on the inflammatory factor TNF‐α were studied, indicating that the microfluidic platform can simultaneously evaluate the cytocompatibility and drug release performance of materials in the same in vitro dynamic system, providing a more physiologically relevant high‐throughput and controllable method for the in vitro functional evaluation of bioceramic materials [116].

In recent years, triply periodic minimal surface (TPMS) structures have received extensive attention in the design of bone tissue engineering scaffolds. TPMS structures have the characteristics of high porosity, excellent mechanical properties, and promoting nutrient transport and can well simulate the structure of natural bone trabeculae. Through additive manufacturing techniques (such as SLS, DLP), bioceramic scaffolds with TPMS structures can be precisely prepared for advanced bone chip platforms.

In bone chips, coculturing TPMS bioceramic scaffolds with osteoblasts, osteoclasts, and endothelial cells can simulate the bone remodeling process and bone–vascular interactions and be used to study pathological processes such as osteoporosis, fracture healing, and bone tumors as well as to test bone‐targeted drugs and bone implant materials.

## 5 Design and Application of Bioscaffolds for Different Organ Chips

**FIGURE 4:**
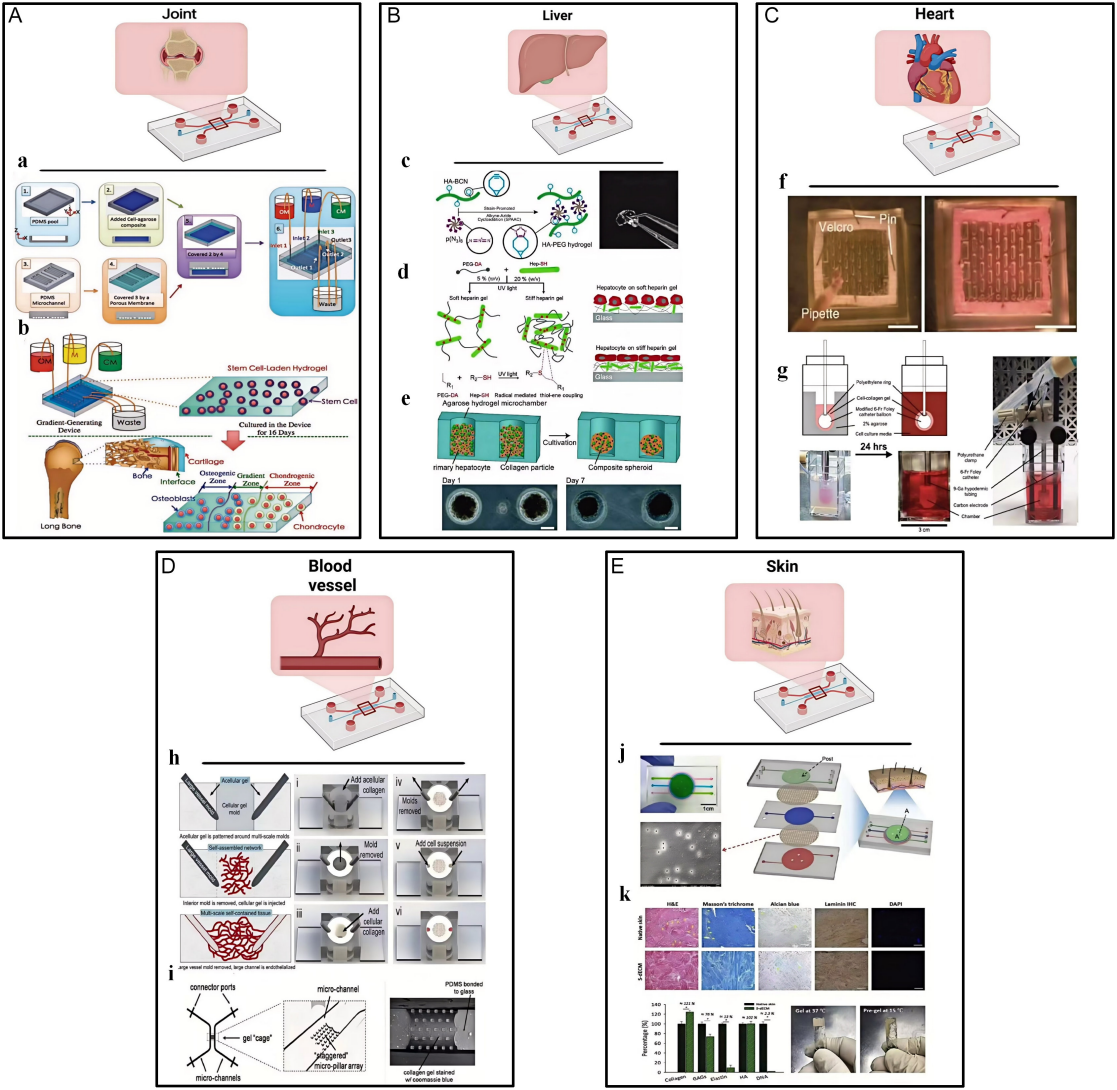
Selection of bioscaffolds for different OoC. (A) Bone OoC. (a) Schematic illustration of the construction of articular cartilage by (a) bio‐microfluidic device using stem cell‐laden hydrogel slab (OM: osteogenic medium, M: normal cell culture medium, CM: chondrogenic medium). (b) Biomimetic structure model of stem cell‐laden hydrogel slab with articular cartilage [117]. Copyright 2012, Wiley. (B) Liver OoC. (c) Schematic diagram and physical photo of the formation of HA‐PEG hydrogel by strain‐promoted alkyne‐azide cycloaddition reaction [118]. Copyright 2018, IOP Science. (d) Heparin gels with different rigidities were prepared by adjusting the concentration of the gel precursor solution and used to culture primary hepatocytes [119]. Copyright 2013, Sage Publications. (e) An agarose hydrogel microchamber with a diameter of 200 μm and a depth of 300 μm is shown, in which cells and particles were cocultured to form aggregates. As the culture time extended, the size of the aggregates gradually decreased, which was caused by the contractile forces between cells–cells and cells–particles. Scale bars, 100 μm [120]. Copyright 2015, Lab on a chip (C) Heart OoC. (f) The cell–hydrogel solution was injected into a PDMS tissue mold through a micropipette and cultured under 37 °C to achieve hydrogel polymerization. Scale bars 5 mm [121]. Copyright 2018, Nature. (g) It shows the construction process, structural schematic diagram, physical morphology of the engineered human ventricular organoid chamber in a customized bioreactor, and the complete device equipped with an electric field stimulation function [122]. Copyright 2018, Elsevier. (D) Blood vessel OoC. (h) It shows the manufacturing process of a multiscale serial perfusion blood vessel structure: first inject cell‐free collagen into the outer ring, inject cell‐containing collagen into the center after removing the internal PDMS struts, then remove the large blood vessel mold, then backfill the channels with endothelial cell suspension, and finally culture [123]. Copyright 2024 American Chemical Society. (i) A device contains two parallel fluid channels and a central gel cage structure, in which staggered microcolumns provide support for the soft hydrogel, can accurately load biological or synthetic matrices, thus isolating the culture medium channels and only allowing molecules to diffuse or convect through the porous matrix [124]. Copyright 2008, Lab on a chip. (E) Skin OoC. (j) Three different colored liquid flows can be seen inside the skin chip device. Its three‐dimensional structure is composed of three layers of PDMS and two layers of PET porous membranes stacked. The PET porous membranes used are from Transwell, with a pore size of about 0.4 μm [125]. Copyright 2016, *Scientific Reports.* (k) It shows the characterization results of skin‐derived dECM bioink, including qualitative analysis of its tissue chemical components by staining (H&E, Masson’s trichrome, Alcian blue, laminin immunohistochemistry and DAPI staining), quantitative analysis of components such as collagen and GAGs, and its sol–gel [126]. Copyright 2018, Elsevier.

This section systematically explains how to select and optimize bioscaffolds based on the physiological structure, mechanical environment, and functional requirements of different organs, using key OoC applications such as joints, the liver, the heart, blood vessels, and the skin as examples (Figure 4). By analyzing the construction examples of each OoC, this section reveals the key role of scaffold materials in simulating organ‐specific microenvironments, summarizes the design logic from material properties to function realization, and provides practical reference and strategic guidance for the development of chips for specific organs.

### 5.1 Bone and Joint Chip

When constructing a bone and joint organ chip, an in‐depth understanding of the physiological structure of the bone matrix is the basis for designing a biomimetic scaffold. The bone matrix is mainly synthesized and secreted by osteoblasts and is remodeled through a continuous process after adulthood. It is a highly ordered composite structure in which type I collagen forms the main organic framework and acts in concert with a variety of noncollagen proteins and hydroxyapatite (HA) crystals deposited on it to endow bones with their characteristic mechanical properties of both rigidity and elasticity [127]. Therefore, it is challenging to simulate the complex mechanical and biochemical microenvironment of bones in a chip, and single materials often struggle to fully reproduce its multidimensional characteristics. Among the numerous hydrogel systems used to support bone formation, those based on collagen or its derivative gelatin are one of the preferred biomimetic materials because of their high similarity to the natural bone collagen matrix in terms of biological origin and their convenience for coinjection with cells. Such hydrogel matrices are often functionally enhanced by incorporating hydroxyapatite or bone‐inductive calcium phosphate crystals to better promote osteogenic differentiation and mineralization processes.

By combining collagen with other hydrogel materials and integrating them into a microfluidic chip, researchers have been able to more comprehensively simulate the biomechanical cues and biochemical signals of natural bones, enhancing the biomimicry and functionality of bone organ chips. For example, scholars such as Roy et al. successfully constructed a chip model simulating the bone marrow niche by coculturing endothelial cells and MSCs in a fibrin–collagen composite hydrogel [128], while Shi et al. utilized microfluidic technology to precisely spatially control the medium gradient in an agarose gel loaded with stem cells, thereby developing a biomimetic osteochondral chip [117].

In joint‐on‐a‐chip, to mimic the multitissue complex structure and function of human joints, multiple interconnected tissue units usually need to be constructed. Among them, cartilage units can use polysaccharide‐based hydrogels. These materials are very similar in composition to the GAG of cartilage matrix. They not only have high biocompatibility, but also can support chondrocytes to maintain their phenotypes and respond to mechanical stimuli, providing a reliable three‐dimensional environment for chondrocytes [129]. However, agarose is difficult to be degraded by chondrocytes, and this property limits the cell migration and remodeling ability in it. Therefore, it is difficult to be used to mimic the angiogenesis process accompanied by osteoarthritis (OA) [130]. In contrast, hyaluronic acid‐based hydrogels have more advantages: chondrocytes can not only interact with this material, but also actively remodel the surrounding ECM through the hyaluronidase expressed by themselves, thus being closer to the dynamic microenvironment under physiological conditions. It is worth noting that under mechanical stimulation, the force distribution and the perception of mechanical signals by chondrocytes highly depend on the physicochemical properties of hydrogel polymers and their crosslinking methods. These factors jointly determine the structural stability and mechanical transmission efficiency of the gel [131]. Therefore, hydrogel systems that can be injected and form gels in situ have attracted much attention in the construction of OoC. Such materials allow the coinjection of polymers, cells, and crosslinkers, with simple operation and high integration efficiency. For example, the dextran–tyramine and hyaluronic acid–tyramine composite system based on enzymatic crosslinking can form stable gels within a few minutes and can be compatibly blended with cells from different sources. It has been confirmed in many studies that it can effectively support the growth and function maintenance of cartilage matrix [132]. Bone units mostly use collagen or gelatin‐based hydrogels and enhance their osteogenic induction and mineralization ability by incorporating functional components such as hydroxyapatite. For example, a composite model was constructed in which chondrocytes differentiated from MSCs were seeded on the surface of a poly(ε‐caprolactone) or its composite scaffold with hydroxyapatite, the scaffold was further embedded in a photocrosslinkable methacrylated gelatin hydrogel, and the hydrogel internally contained endothelial cells and MSCs to mimic the microenvironment of bone tissue [133]. The synovial unit needs to mimic its bilayer structure and usually realizes the coculture of synovial fibroblasts and immune cells through chambers separated by porous membranes. The material selection focuses on supporting cell adhesion and migration; due to the layered structure of the meniscus unit, two hydrogels with different elastic modulus and composition are often used to mimic the vascularized outer layer and the fibrocartilage inner layer respectively; ligament units mostly use collagen‐based hydrogels containing fibroblasts and simulate their anisotropic mechanical properties through fiber prealignment; the Hoffa fat pad unit is generally constructed with a three‐dimensional vascularized hydrogel containing adipocytes. Generally speaking, the scaffold selection of each tissue unit in joint‐on‐a‐chip needs to take into account the material biological properties, mechanical properties, and the construction of tissue‐specific microenvironment to support the realization of cell differentiation, tissue communication, and dynamic mechanical response.

In recent years, the construction of in vitro OA models using OoC technology has provided a new platform for drug screening. For example, Zhao et al. developed a chondrocyte chip system for OA drug evaluation [134]. In this system, primary chondrocytes were encapsulated in methacrylated gelatin (GelMA) hydrogel and combined with microfluidic technology to dynamically simulate the physiological microenvironment of cartilage tissue. This platform can not only maintain the phenotypic stability of chondrocytes but also successfully induce an OA‐like inflammatory state through interleukin‐1β (IL‐1β) treatment. Studies have shown that this model exhibits highly consistent responses to nonsteroidal anti‐inflammatory drugs (NSAIDs), the TRPA1 channel inhibitor HC‐030031, and exosomes derived from Lactobacillus rhamnosus (LGG‐EVs), verifying its feasibility and reliability in OA drug screening.

Due to the excellent drug loading and slow‐release properties of HMs, they have received extensive attention in the treatment research of OA. Shen et al. used microfluidic technology and self‐assembly technology to prepare silk fibroin–DNA HMs with excellent biocompatibility and elastic modulus [135]. These microspheres with RGD peptide sequences on their surfaces significantly enhanced cell adhesion and promoted the in vitro differentiation of bone marrow MSCs (BMSCs) into chondrocytes, successfully creating cartilage organoid precursors (COPs). Further in vivo studies showed that loading BMSCs into RSD‐MS microspheres significantly promoted cartilage repair, and the repair effect was better than that of the group using only silk fibroin–DNA HMs (SD‐MS).

### 5.2 Liver Chip

As the core metabolic organ of the human body, the liver undertakes various key physiological functions, including serum albumin synthesis, energy metabolism, drug transformation, and detoxification. The maintenance of its functions highly depends on the complex homotypic and heterotypic cell interactions between hepatocytes and nonparenchymal cells (such as Kupffer cells, stellate cells, etc.), and these interactions are crucial for maintaining liver‐specific phenotypes and functions in vitro. Therefore, constructing an in vitro model that can simulate the physiological structure of the liver is of great significance. Among them, the liver chip, as an advanced in vitro liver model, has shown important application value in fields such as drug screening and hepatotoxicity assessment.

The key to in vitro liver model engineering lies in developing scaffolds/matrices that can fully simulate the liver microenvironment to support hepatocytes to achieve real functional expression. As the physical support for hepatocyte growth and function maintenance, the performance of the scaffold is affected by various factors such as porosity, pore size, biomechanical properties, and its structural design.

PEG hydrogels are widely used in the engineering of liver microtissues in liver‐on‐a‐chip due to their high permeability, biocompatibility/immunological inertness, and the presence of acrylic ligand functionality. In a previous study, researchers developed a modular hydrogel based on hyaluronic acid‐polyethylene glycol (HA‐PEG) and used it for the three‐dimensional culture of human‐induced pluripotent stem cell‐derived hepatocytes (hiPSC‐HEPs) in a dynamic liver‐on‐a‐chip system by coupling with RGD peptides. Results showed that this hydrogel system significantly promoted the formation of three‐dimensional liver‐like tissues while improving cell viability and albumin synthesis levels [118]. Therefore, the introduction of bioactive cell adhesion sequences (such as RGD peptides) can be regarded as an effective strategy to optimize hydrogel systems and promote the development of biomimetic liver‐on‐a‐chip platforms. To reproduce the microenvironment of the natural ECM, the design and biomechanical properties of the substrate are particularly important. Therefore, researchers increasingly adopt biomimetic strategies to mimic the liver structure in healthy or diseased states [136]. Hepatocytes are prone to loss of function during high‐density culture, while hydrogel scaffolds can provide three‐dimensional support to effectively maintain the function and viability of hepatocytes. For example, the collagen bilayer structure has been widely used as a classic two‐dimensional hepatocyte “sandwich” culture model [137]. On this basis, improved biomaterials have emerged, such as collagen‐incorporated poly(lactic‐co‐glycolic acid) (PLGA) scaffolds, which significantly improve hepatocyte survival and functional performance due to their enhanced bioactivity [138, 139]. Similarly, natural polymers such as silk fibroin modified with the arginine‐glycine‐aspartic acid (RGD) sequence can promote the formation of functional hepatocyte clusters [140]. RGD modification also facilitates the adhesion of liver sinusoidal endothelial cells (LSEC) and enables endothelialization of the material surface [141]. Regarding highly porous hydrogel scaffolds, research reports have shown that scaffolds composed of alginate and galactose‐modified chitosan can support the efficient culture of hepatocyte spheroids [142]. In addition, scaffolds based on synthetic polymer films can achieve a spatially ordered coculture system of hepatocytes and nonparenchymal cells [143].

In recent years, as understanding of mechanical signaling has deepened of the mechanism of mechanical signal transduction, researchers have begun to focus on developing scaffold materials that can mimic the liver elastic modulus under physiological and pathological conditions. For example, heparin hydrogels can regulate the function of hepatocytes by adjusting their elastic modulus: the albumin secretion of hepatocytes cultured in softer (10 kPa) hydrogels is significantly higher than that of cells cultured under stiffer (110 kPa) conditions after 5 days [119]. Experiments on polyacrylamide gels have further confirmed that as the matrix elastic modulus increases, the activity of primary metabolic enzymes decreases, while the ability of albumin secretion and urea synthesis is significantly enhanced, thus more accurately predicting drug‐induced liver toxicity [144, 145].

In the process of constructing a bionic liver model, HMs have become a highly promising cell carrier in liver‐on‐a‐chip due to their ability to mimic the three‐dimensional structure of the ECM, provide mechanical support, and allow the diffusion of nutrients and signaling molecules. These microspheres can better maintain hepatocyte polarity, promote the expression of liver‐specific functions, and contribute to the realization of physiological cell–cell and cell–matrix interactions, thus enhancing the biological relevance of the chip model and the predictive value of drug evaluation. Yamada et al. [120]. developed a method based on the combination of microfluidics and membrane emulsification technology to successfully prepare cell‐sized collagen microspheres. This method achieved efficient control of the microsphere size (5–20 μm) and its flattened morphology by rapidly extracting and concentrating the collagen solution. The obtained microspheres have good cell affinity and can be coassembled with primary rat hepatocytes in a nonadhesive hydrogel microchamber to form composite cell spheres. Thanks to their bionic biochemical and structural properties, these HMs significantly increased the expression levels of specific genes in hepatocytes, providing a feasible strategy for precisely mimicking cell–cell and cell–matrix interactions in a three‐dimensional culture system.

### 5.3 Heart Chip

The heart is a vital organ composed of cardiomyocytes (CMs), responsible for pumping blood throughout the body, thereby transporting oxygen, nutrients, and metabolic wastes in the blood vessel network. In recent years, heart‐on‐a‐chip systems have been developed to study heart morphology, function, and drug responses.

In cardiac tissue engineering, hydrogel scaffolds such as Matrigel, fibrin, collagen, and synthetic methacrylated gelatin (GelMA) are widely used, mainly because their soft mechanical properties can mimic the biomechanical environment of the native ECM. In this biomimetic microenvironment, CMs can establish appropriate cell–cell contacts and secrete their own ECM, thus enabling a three‐dimensional culture closer to the physiological state. For example, researchers encapsulated human embryonic stem cell‐derived CMs in a collagen/Matrigel hybrid hydrogel and used a 3D printing mold to construct three‐dimensional cardiac strips [121]. Spontaneous beating appeared on the 3rd day of culture in this cell–hydrogel complex, indicating that the porous structure of the hydrogel provided the necessary microenvironmental support for cell survival [146]. Further, mechanical loading, especially cyclic stretching, has been shown to contribute to the construction of more mature cardiac microtissues. Compared with the static stretching or nonstretched control groups, the constructs subjected to mechanical stimulation exhibited enhanced contractility, elastic modulus, sarcomere length, and related molecular expression characteristics, thus overall improving the mechanical function of the microtissues. In addition, Sidorov et al. [147] cocultured neonatal rat ventricular CMs (NRVMs) with a fibrin/Matrigel hybrid hydrogel in a PDMS mold, integrated it into a six‐well plate, and placed it on an electromechanical microscope platform equipped with a camera. By calibrating the lateral displacement of a flexible probe, progressive strain loading was applied to the three‐dimensional cardiac tissue, and the deformation response of the construct was synchronously captured by the camera. The results showed that under controllable mechanical stimulation, the CMs were longitudinally arranged and the sarcomere structure was more developed, with a significant increase in the contraction amplitude. The construct also showed the expected responses to β‐adrenergicstimulation and myosin inhibitors, providing an effective platform for cardiac mechanism research and drug screening.

Another approach to constructing engineered heart structures is to mix collagen with ventricular CMs derived from human pluripotent stem cells (hPSCs), shape it around a balloon catheter, and form a structure with a hollow chamber after 10 days of culture. This tissue exhibits a pumping function similar to that of the natural heart and can quantitatively evaluate the relationship between stroke volume and end‐diastolic volume through pressure development and volume changes, thus enabling the in vitro characterization of the heart’s pumping characteristics [122]. In summary, these methods all utilize hydrogel materials as scaffolds to construct a biomimetic heart tissue engineering platform, providing an important tool for studying CM–ECM interactions and the relationship between heart structure and function. However, in most current construction processes, cells are simply mixed into the hydrogel without an external guiding structure, resulting in a chaotic cell arrangement and difficulty in reproducing the multilayered ordered structure of natural myocardium. Therefore, although hydrogel scaffolds demonstrate excellent support for biomimetic microenvironments in three‐dimensional culture, how to achieve a high degree of organization and structural stratification of CMs based on this remains the main challenge in this field.

Synthetic polymer materials have also become important material choices in the field of tissue engineering. Among them, polyester materials show broad prospects due to their physiologically matched adjustable elastic modulus, excellent cyclic tensile resistance, appropriate short‐term and long‐term degradation rates, ease of processing, and potential for surface modification [148]. These materials can be hydrolytically degraded into monomers such as glycerol or citric acid that can be naturally metabolized by the body, thus having good potential in transplantation applications. In cardiac tissue engineering, the Young’s moduli of high‐elastic‐modulus polyesters such as polycaprolactone (PCL) and poly(lactic‐co‐caprolactone) (PLCL) reach 3.8 ± 0.8  and 8.4 ± 0.9 MPa, respectively, and their mechanical properties are often regulated by techniques such as solvent casting or electrospinning [149]. An OoC microdevice engineering method based on synthetic polymer elastomers proposed by Lai et al. can prepare microchannels that can serve as independent biocompatible scaffold templates, provide stability for the perfusable vascular network, and promote the remodeling of dense three‐dimensional parenchymal tissues; this method enables the convenient integration of organoids in the chip system by spatially separating the vascular and tissue systems and controlling limited fluid transfer [150].

### 5.4 Vascular Chip

In the research and application of vascular OoC, hydrogels are key materials for constructing three‐dimensional vascular‐like structures [151]. A commonly used strategy is to fill the microchambers or microchannels of the chip with natural hydrogels (such as Matrigel, collagen, fibrin/fibrinogen, and dECM, etc.) and culture endothelial cells in this three‐dimensional environment to enable self‐assembly and spontaneous blood vessel formation [151]. This method provides a highly biomimetic and controllable alternative for generating functional and perfusable microvascular networks in vitro and studying the angiogenesis process. For example, coculturing endothelial cells and pericytes in fibrin or collagen hydrogels in a microfluidic environment can form vascular networks with well‐defined lumen structures, providing an effective in vitro model for studying angiogenesis, tumor metastasis, and vascular‐related diseases. The initial process of angiogenesis (i.e., tubulogenesis) has been directly simulated in a gel cell culture system by embedding endothelial cells in a remodelable three‐dimensional matrix (such as collagen, fibrin, or Matrigel) [152, 153]. After embedding in such ECM hydrogels, endothelial cells can form lumen‐like structures through intracellular or extracellular mechanisms and further construct interconnected vascular networks [152]. A large number of studies have used this direct gel culture system to reveal the key factors and conditions that guide endothelial cells to form tube‐like structures and connect and remodel according to biophysical and biochemical signals [154, 155]. In addition, surrounding cells (such as pericytes) can also be embedded in the overall gel together, providing structural support for developing blood vessels by promoting the assembly of basement membrane matrix [156].

HMs offer theoretical promise for vascular OoC due to their porous structure, which mimics the perivascular ECM and supports endothelial cell adhesion and nutrient diffusion. They can also function as vehicles for targeted delivery of cells or bioactive molecules. Nevertheless, direct experimental evidence of their integration into functional, perfusable vascular networks in OoC remains scarce, making this an underexplored but promising direction.

To construct a perfusable and structurally emulative vascular network model, researchers have developed various integrated strategies based on microfluidic technology. For example, Donzanti et al. proposed a simple and efficient method for constructing a vascular network with multiscale characteristics and continuous perfusion [123]. By combining cell self‐assembly and micromolding techniques, this system embedded self‐generated small‐scale capillary beds into deterministically processed large‐scale vascular channels, achieving tight connection and fluid communication between different‐scale blood vessels while avoiding the common leakage problems in traditional microvascular models. The system also established a clear perfusion path, prompting fluid to flow from the inlet through the capillary bed to the outlet. The research further verified the connectivity and fluidity of this multiscale network by perfusing fluorescent microspheres.

Adopting a similar concept, Vickerman et al. designed a PDMS microfluidic platform with a “gel cage” formed by a microcolumn array in the center for precise injection and mechanical fixation of biohydrogels (such as collagen or synthetic polypeptide gels) [124]. Using this platform, researchers were able to stably culture human microvascular endothelial cells (HMVECs) in a three‐dimensional hydrogel and flexibly regulate biochemical gradients, shear stress, and matrix flow in the microenvironment while supporting real‐time imaging of cell behavior. By adjusting the fluid pressure difference and culture conditions, this platform successfully induced the formation of capillary‐like structures, which was confirmed by fluorescence microsphere perfusion and confocal microscopy observation. In addition, this platform is compatible with various three‐dimensional angiogenesis models (such as single‐cell encapsulation, matrix invasion, and two‐dimensional migration) and successfully captured dynamic processes such as filopodia extension, cell polarization, division, and lumen formation, thus providing a structurally controllable and functionally diverse in vitro analysis tool for studying angiogenesis mechanisms and cell interactions.

In summary, successful vascularization remains a critical determinant for the physiological function of most OoC models. Current strategies include: (i) self‐assembly of endothelial cells in hydrogels (e.g., collagen, fibrin), (ii) sacrificial templating (e.g., 3D‐printed fugitive inks), (iii) microfluidic channel embedding, and (iv) modular assembly of vascularized microtissues. Future efforts should focus on generating perfusable, hierarchically branched networks that can be easily integrated with parenchymal compartments. Combining these approaches with dynamic perfusion and coculture of pericytes or fibroblasts will further improve vessel maturation and barrier function.

### 5.5 Skin on a Chip

The human skin is an important physiological barrier of the body, protecting internal tissues and organs from harmful stimuli such as external pathogens, ultraviolet radiation, and environmental pollutants. As an organ with a complex structure, the skin consists of multiple cell layers (such as the epidermis, dermis, and subcutaneous tissue) and rich accessory structures (such as blood vessels, hair follicles, and sweat glands) and has various physiological functions such as tactile perception and temperature regulation. In recent years, in vitro three‐dimensional skin models (such as skin‐on‐a‐chip) have gradually become an important alternative to traditional monolayer cell culture, which can better simulate the multilayer anatomical structure and physiological functions of the human skin and show broad application prospects in fields such as pathology research, drug development, and assessment of the safety and efficacy of cosmetics.

When constructing skin‐on‐a‐chip, hydrogels, as the supporting matrix or three‐dimensional culture carrier, have become the key materials for constructing bionic skin models with complete structures and functions by mimicking the ECM and providing a compartmentalized microenvironment. Among them, the selection of biomaterials for the dermal scaffold is particularly crucial, as it needs to promote cell–matrix interactions and nutrient exchange while maintaining sufficient mechanical stability to resist matrix deformation and degradation caused by fibroblast proliferation and active contraction [157]. Since collagen is the core component of the skin ECM, it has become the preferred natural material for constructing the dermal matrix. However, pure collagen hydrogels have weak elastic modulus and are prone to rapid degradation, shrinkage, and detachment from the substrate during culture, which is also a problem in commonly used natural hydrogels such as fibrin [158]. Matrix shrinkage not only shortens the lifespan of engineered skin models but also hinders their standardization and application promotion [159].

To overcome the above limitations, researchers often improve the mechanical properties of natural hydrogels through crosslinking or material composite strategies. Although chemical crosslinking agents (such as glutaraldehyde and genipin) can effectively enhance the stability of the scaffold, their potential cytotoxicity needs to be carefully evaluated. Therefore, developing crosslinking methods with better biocompatibility has become an important direction. For example, using nontoxic PEG‐based crosslinking agents (PEG‐SG) can keep the collagen hydrogel structurally stable during long‐term culture and significantly inhibit shrinkage [160]. In addition, natural materials such as hyaluronic acid and gelatin can also be functionalized and modified with methacrylic anhydride to form photocrosslinkable hydrogels, thereby achieving fine regulation of their mechanical properties and further combining with bioactive molecules (such as dopamine) to enhance their wound healing and angiogenesis‐promoting abilities [161].

In the actual construction of skin chips, the optimized hydrogel matrix has shown good application potential. For example, Sriram et al. combined a nonshrinking fibrin dermal matrix with a dynamic microfluidic system to successfully construct a full‐thickness skin chip model. This model showed better epidermal differentiation and barrier function under flow culture conditions, with stable structure and convenience for downstream assays such as transdermal absorption, providing a reliable platform for drug screening and skin toxicology research [162].

Skin chips provide an important in vitro platform for preclinical drug screening, toxicology research, and exploration of skin pathological mechanisms. For example, a study used a porous membrane coated with fibronectin to construct a skin chip with a typical stratified structure, successfully simulating the barrier function of the skin [125]. This model further simulated the states of inflammation and edema by introducing tumor necrosis factor‐α (TNF‐α) into the dermis layer, thus being used for the efficacy evaluation of anti‐inflammatory drugs. In addition, skin equivalent models are also effective tools for studying wound closure and skin regeneration, especially suitable for the repair of large‐area wounds that are difficult to heal spontaneously.

In recent years, with the development of organ chip technology, it has been possible to construct skin equivalents in systems with physiologically relevant structures and functions. For example, in a study, a prevascularized skin model was constructed using skin‐derived ECM bioink through 3D bioprinting technology [126]. After transplantation of this skin patch to the skin defect site of mice, it significantly promoted the processes of wound closure and re‐epithelialization, verifying the feasible potential of such hydrogel‐based skin models in regenerative medicine and clinical translation.

## 6 Challenges and Future Outlook

### 6.1 Main Challenges Currently Faced

Although OoC and bioscaffold materials have shown great potential in tissue engineering and drug development, their further development is still limited by multiple challenges such as lack of standardization, difficulty in long‐term function maintenance, insufficient vascularization, complexity of multiorgan integration, and high cost. Looking to the future, through interdisciplinary collaborative innovation and key technology research, this field is expected to achieve breakthroughs in the following directions. 1. Standardization and reproducibility: There are significant differences in the scaffold materials and manufacturing processes used in different laboratories, making it difficult to compare and replicate results. It is necessary to establish a standardized evaluation system and quality control standards to ensure the reliability and comparability of experimental results. 2. Long‐term stability and function maintenance: Currently, most OoC can only maintain tissue function for a short period (usually a few weeks), making it difficult to achieve long‐term steady‐state culture. It is necessary to develop more stable scaffold materials and culture systems to support the long‐term maturation and function maintenance of tissues. 3. Vascular network integration: Effective vascularization is a key challenge in simulating the functions of most organs. Although there are various strategies (such as spontaneous angiogenesis, sacrificial material pore formation, 3D printing of blood vessels), it is still difficult to achieve efficient integration with host blood vessels and functional perfusion. 4. Complexity of multiorgan coupling: Connecting multiple organ chips to simulate whole‐body responses faces challenges in scale matching (e.g., relative sizes, cell numbers), flow rate coordination (e.g., different perfusion requirements per organ), and crosstalk (e.g., metabolite interference, signaling crosstalk). Standardized interface designs, microfluidic circuit analogies (e.g., resistors, capacitors), and computational modeling are needed to ensure physiologically relevant multiorgan interactions. Furthermore, the integration of a functional immune component across organs remains a major hurdle. 5. Cost, accessibility, and regulatory hurdles: Many advanced scaffold materials and fabrication techniques remain expensive, limiting widespread adoption. Moreover, the path to clinical translation is obstructed by the lack of regulatory qualification standards for OoC devices. To date, only the Emulate liver chip has received FDA qualification for DILI. Bridging this gap will require collaborative efforts between academia, industry, and regulatory agencies to establish validation protocols and quality control benchmarks. 6. Lack of dynamic and reversible scaffold systems: Most existing scaffolds are static and cannot be remodeled or removed on demand. There is a notable gap in the systematic development and application of dynamic scaffolds that can respond to biological cues (e.g., enzymes, pH) or external stimuli (e.g., temperature, light) or be reversibly liquefied for noninvasive cell retrieval and matrix remodeling studies. Emerging materials such as thermoreversible PIC hydrogels represent a promising direction, but their integration into OoC remains largely unexplored. 7. Recapitulation of functional immune responses: The lack of a fully functional immune system in most current OoC models significantly limits their applicability in toxicity testing, vaccine development, and immunomodulatory drug screening. Integrating immune cells (e.g., macrophages, T cells) into OoC platforms remains challenging due to their dynamic behavior, migration, and crosstalk with parenchymal cells. Furthermore, scaffold materials themselves can trigger innate immune responses, as discussed in Section 4.1.2 , complicating data interpretation. Future efforts should focus on developing standardized immunocompetent OoC models with well‐characterized scaffold immunogenicity.

### 6.2 Future Development Trends

To address the above challenges, the future development of bioscaffolds in OoC will no longer be limited to the improvement of materials themselves, but will show a systematic innovation trend of deep interdisciplinary integration. Looking ahead, the research directions will focus on the following key areas, aiming to build a next‐generation OoC system with more complete functions, richer information, and more precise applications. 1. Personalized and precision medicine: Combining patient‐specific iPSC technology and personalized scaffold design to construct disease‐specific models for personalized drug screening and treatment optimization. The data‐driven framework will support personalized scaffold design based on medical imaging and functional test results, enabling true precision medicine. 2. Smart‐responsive and dynamic scaffolds: Developing scaffold materials that can dynamically respond to biological signals (e.g., enzymes, pH) or external stimuli (e.g., light, magnetism, temperature) will enable on‐demand control over cell behavior, drug release, and scaffold remodeling. Thermoreversible hydrogels (e.g., PIC), photocleavable gels, and enzyme‐degradable crosslinkers are prime examples. Integration of such dynamic scaffolds into OoC will significantly enhance the physiological relevance and experimental versatility of the platform. 3. Multimaterial and gradient design: Using multimaterial 3D printing and gradient manufacturing techniques to create heterogeneous scaffolds with spatially varying composition, structure and properties, better simulating tissue interfaces, and the microstructure of complex organs [163]. 4. Integrated sensing and real‐time monitoring: Integrating microsensors (such as temperature, pH, oxygen partial pressure, glucose sensors) into the scaffold to real‐time monitor microenvironment parameters and cell functions, providing more comprehensive dynamic data [164]. 5. Green manufacturing and sustainable development: Developing environmentally friendly manufacturing processes and recyclable materials to reduce the use of organic solvents and energy consumption and reducing the environmental footprint of OoC technology. 6. Organoid and chip integration: Combining organoid technology with OoC and leveraging the self‐organizing ability of organoids and the precise control advantages of chips to create more physiologically relevant tissue structures and functions [165]. 7. AI‐driven intelligent design and system integration: Introducing artificial intelligence as a cross‐functional enabling tool throughout the entire process of scaffold development and application [166, 167, 168]. This specifically involves utilizing deep learning algorithms to analyze multimodal data generated by OoC/organoid systems, enabling intelligent recognition of cellular behavior and prediction of drug responses [166].

## 7 Conclusion

OoC technology holds promise as a powerful tool for biomedical research and drug development, but its actual impact on these fields will depend on overcoming current limitations in standardization, vascularization, long‐term stability, and regulatory acceptance. Among them, the bioscaffold, as the support basis for cell growth and tissue formation, plays a decisive role in the performance of the chip. This article systematically reviews the selection and application strategies of bioscaffolds in OoC, with a focus on the characteristics, applications, and optimization strategies of hydrogels, HMs, and other types of scaffolds. Among them, the bioscaffold, as the support basis for cell growth and tissue formation, plays a decisive role in the performance of the chip. This article systematically reviews the selection and application strategies of bioscaffolds in OoC, with a focus on the characteristics, applications, and optimization strategies of hydrogels, HMs, and other types of scaffolds.

Hydrogel scaffolds, with their high water content, properties similar to natural ECM, and good biocompatibility, have become the most commonly used scaffold materials in OoC, especially suitable for simulating soft tissues such as the liver, kidney, and brain. HMs, with their large specific surface area, injectability, and controllable release characteristics, have shown unique advantages in dynamic culture and drug delivery, providing new ideas for creating more complex microenvironments. The mechanical properties and controllable degradation of other scaffold materials such as synthetic polymers, the osteoconductivity of bioceramics, and the synergistic effects of composite materials all have irreplaceable values in specific applications.

Data‐driven frameworks combined with advanced manufacturing technologies are changing stent design and selection strategies, enabling the customization of personalized stents and optimizing the performance and predictability of OoC. However, the field still faces challenges such as standardization, vascularization, long‐term stability, and multiorgan coupling and requires interdisciplinary collaboration and innovative solutions.

In the future, with the continuous development of materials science, microfabrication technology, and artificial intelligence, the bioscaffolds in OoC will become more intelligent, personalized, and functional, providing a more powerful platform for drug development, disease modeling, and personalized medicine. Through continuous innovation and interdisciplinary cooperation, OoC technology is expected to ultimately reduce the use of animal experiments, accelerate the clinical translation of treatment methods, and promote the development of precision medicine.

## Author Contributions

All authors contributed to the manuscript and approved the final version.

## Funding

This study was supported by Center for Neuromusculoskeletal Restorative Medicine, CUHK Peter Hung Pain Research Institute (PHPRI/2024/122), National Natural Science Foundation of China (82302753), Hong Kong Research Grants Council (24203523), Mainland‐Hong Kong Joint Funding Scheme, Innovation and Technology Commission (#MHP/101/23), and Guangdong‐Hong Kong Technology Cooperation Funding Scheme (#GHP/140/22GD).

## Conflicts of Interest

The authors declare no conflicts of interest.

## Data Availability

Data sharing not applicable to this article as no datasets were generated or analyzed during the current study.
